# Sex Differences in Spatial Cognition Extend Beyond Vision: Insights From the Audio‐Corsi Test

**DOI:** 10.1111/ejn.70226

**Published:** 2025-08-12

**Authors:** Daniela E. Aguilar Ramirez, Jennifer Kane, Walter Setti, Lara Coelho, Monica Gori, Claudia L. R. Gonzalez

**Affiliations:** ^1^ Department of Kinesiology and Physical Education University of Lethbridge Lethbridge Alberta Canada; ^2^ U‐Vip Unit Italian Institute of Technology Genoa Italy

**Keywords:** auditory spatial tasks, cognitive strategies, mental rotation, Sex differences, spatial abilities, spatial working memory

## Abstract

Over the past several decades, substantial evidence has accumulated demonstrating sex differences in spatial abilities. Males outperform females in most visual tasks that require processing visuospatial information. Notably, in real‐world contexts, this capacity also involves other sensory modalities, such as the auditory system. However, unlike visuospatial abilities, research on sex differences in auditory spatial abilities remains sparse. The present study investigated sex differences in an auditory spatial working memory task. Seventy‐seven participants (41 female) completed the Audio‐Corsi task, the well‐established visual Mental Rotation Test (MRT), and a cognitive strategy questionnaire. Results revealed that males outperformed females on both the Audio‐Corsi and the MRT. Interestingly, a significant relationship between performance on the Audio‐Corsi and the MRT emerged, but only among females. Furthermore, to complete the Audio‐Corsi, males reported employing the use of a holistic cognitive strategy more than females. These findings demonstrate that sex differences in spatial abilities extend across sensory modalities, encompassing both auditory and visual domains. They also underscore the distinct cognitive strategies employed by males and females in spatial processing. This study contributes to a deeper understanding of sex differences in spatial cognition.

## Graphical abstract

Sex differences in spatial cognition extend beyond vision. Sex differences in spatial abilities are well established using vision. But are there sex differences in spatial abilities using audition? Mental Rotation Task‐Auditory Spatial Task‐Questionnaire. Dependent Variables are as follows: MRT score, sequences (1–36), span (2–9), total score (seq*span). Training phase followed by the forward condition (recall of sequences in the same order) and backward condition (REVERSE order). Males performed better than females in the auditory spatial task. The relationships between auditory and visuospatial tasks are sex‐specific.

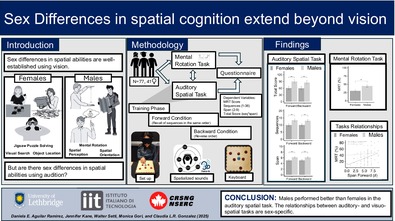

**Abbreviations**
ANOVA: analysis of variance
CBT: Corsi block tapping
IBM: International Business Machines Corporation
MATLAB: Matrix Laboratory
MRT: Mental Rotation Test
SPSS: Statistical Package for the Social Sciences
USB: universal serial bus

## 1 Introduction

In humans, the brains of females and males have evolved distinct structural and functional characteristics. Differences in brain metabolic activity, anatomy, and functional connectivity between the sexes have been well documented (Cosgrove et al. 2007; Halpern 2011; Kolb and Whishaw 2021; Lee et al. 2024). These differences in brain structure and function have been associated with observable sex differences in cognitive ability (Halpern 2011; Kolb and Whishaw 2021). Performance differences are found in spatial, verbal, and memory abilities (among others). Across cultures and age groups, sex differences in cognition are most prominent and consistently observed in spatial abilities (Voyer et al. 1995; Lippa et al. 2010; Levine et al. 2016; Lauer et al. 2019; Aguilar Ramirez et al. 2020; Newcombe 2020). Spatial abilities refer to the human capacity to acquire, understand, remember, organize, and use spatial information about the environment. This process is accomplished through the integration of inputs from all sensory systems, visual, auditory, and haptic modalities (Halpern 2011; Kolb and Whishaw 2021). However, most of the research on sex differences in spatial abilities has primarily focused on the visual domain. Numerous studies have demonstrated that males tend to outperform females on a variety of visuospatial tasks (Castro‐Alonso and Jansen 2019). These include the Water Level Test (Inhelder and Piaget 1958), the Rod‐and‐Frame Test (Witkin et al. 1962), the Paper Folding Test (Ekstrom et al. 1976), and the Embedded Figures Test (Witkin 1971). Among these, the most pronounced sex difference is consistently observed in performance on the Mental Rotation Test (MRT; Shepard and Metzler 1971). The MRT is a paper‐based task in which participants are required to mentally rotate three‐dimensional figures and to identify those that match a target figure.

Despite the robust evidence of sex differences in spatial abilities within the visual modality, relatively few studies have explored such differences across other sensory domains. In the present study, we use a paradigm developed by Setti et al. (2021) to investigate sex differences in auditory spatial cognition. The task is based on the visual spatial working memory test developed by Corsi (1972). The Corsi block tapping (CBT) test consists of presenting the participant with an array of nine wooden blocks; the experimenter taps the blocks in a sequence, and the participant recalls the sequence and taps it back in the same or in reverse order of presentation. Sequences of increasing length are tapped (starting with two blocks and up to nine blocks) until the participant reaches their limit (memory span). Corsi (1972) found that patients with right‐temporal lobe removal were impaired at the task; patients could not learn new sequences or learned them very slowly. Since then, the CBT has been widely used to assess spatial memory in patients or normal populations, as well as to examine individual differences. Using this task, studies have shown a male advantage in the visual CBT (Voyer et al. 2017) consistent with the literature showing a male advantage in other visuospatial processes. Setti et al. (2021) adapted the CBT to the auditory domain, the Audio‐Corsi. In this auditory version, participants are asked to remember sequences of six spatially localized sounds; the sequences also increase in length (from two and up to nine sounds). Setti et al. compared performance between vision and audition. Their findings revealed better performance in the visual when compared to the auditory domain. They suggested that this may be due to the different roles these two sensory systems play in spatial location, with vision providing more spatial resolution than audition. Importantly, they found the Audio‐Corsi test to be a reliable task to measure audio‐spatial memory abilities.

Although Setti et al.'s study provided a valuable foundation for assessing auditory spatial memory, it did not examine potential sex differences in performance. More broadly, research on sex differences in auditory spatial cognition remains sparse and has primarily concentrated on auditory spatial localization rather than memory. Spatial localization paradigms often involve presenting multiple simultaneous sounds from different locations and requiring participants to focus on one sound and determine its position. For instance, males have shown an advantage in tasks such as perceiving approaching sound motion (Neuhoff et al. 2009), vertical sound localization using the right ear in monaural conditions (Lewald 2004), and detecting increased‐bandwidth oddball targets originating from speakers on the participant's left or right side (Simon‐Dack et al. 2009). Together, these findings suggest that males tend to outperform females in auditory spatial localization tasks. However, auditory spatial memory, the ability to temporarily store and recall spatial information about sounds, serves a different and equally critical function. This ability allows individuals to navigate dynamic environments safely and effectively. Whether walking, driving, or crossing a street, we must keep track of auditory cues such as car engines, sirens, alarms, or conversations. Without this capacity, everyday functioning would be significantly compromised. Despite the importance of this ability, no prior studies, to our knowledge, have specifically investigated sex differences in auditory spatial memory. As such, it remains unclear whether the male advantage observed in auditory spatial localization tasks extends to spatial memory. The current study, therefore, investigates sex differences in auditory spatial memory using the Audio‐Corsi task. Due to the limited research in this area, we also included the well‐known MRT as a validation measure to confirm that our participants' results align with findings from previous studies on visuospatial cognition. We hypothesized that males would score higher in the Audio‐Corsi and the MRT and that participants with better performance in one task would also exhibit better performance in the other task. Finally, we included a comprehensive questionnaire examining possible strategies used by females and males.

Prior research suggests that females tend to approach visuo‐spatial tasks using an analytic/detailed strategy, whereas males often adopt a global or holistic approach to such tasks (Pletzer 2014). Although these strategy differences may contribute to sex‐related variation in visuo‐spatial tasks, it remains underexplored if these strategies also play a role in the auditory‐spatial domain.

## 2 Methods

### 2.1 Participants

Seventy‐seven participants between the ages of 17 and 36 years old (M = 21.4, SD = 3.9, Mdn = 20) volunteered for the study. These participants self‐reported their sex (Male, Female, Prefer to self‐describe as________, Prefer not to answer) and gender (Woman, Man, Prefer to self describe_________, Prefer not to answer). Our sample consisted of 41 female and 36 male participants, and all participants self‐identified as cis‐gender.

A sensitivity power analysis was conducted using G*Power 3.1 (Faul et al. 2009), which suggested that with 77 participants, a 2 condition (forward and backward) by 2 sex (female and male) repeated measures ANOVA could detect an interaction with a small effect size (*f* = 0.13) with 0.80 power (*α* = 0.05, *r* = 0.50, and *ϵ* = 1). Participants were all healthy University of Lethbridge undergraduate students, who received course credit for their participation. The participants completed the task with their right dominant hand and did not have any hearing impairments as per recruitment conditions (i.e., to be right‐handed and without visual or auditory impairments). They were recruited through the Department of Psychology, using participant management software (Sona Systems). The experiment was approved by the University of Alberta Research Ethics Board 2.

### 2.2 Tasks

#### 2.2.1 Audio‐Corsi

**FIGURE 1:**
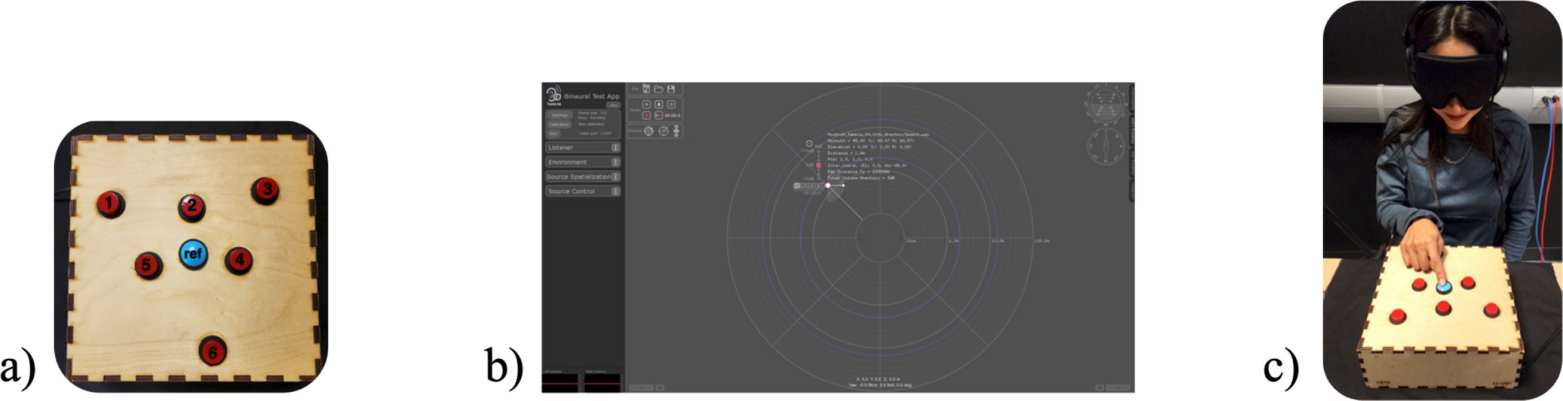
The Audio‐Corsi Task. (a) the Arduino‐based keyboard. (b) The 3D Tune‐In Toolkit Binaural Test App (https://www.3d‐tune‐in.eu). (c) The example of Audio‐Corsi setup.

**FIGURE 2:**
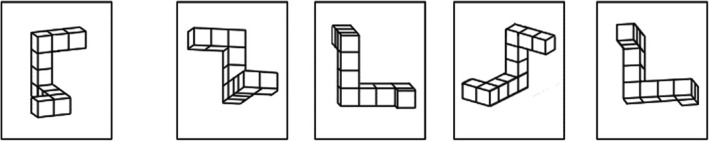
The Mental Rotation Test. *Note:* one trial of the Mental Rotation Test (MRT). The target figure on the left is compared to the four figures on the right. The participant attempts to identify the two figures that are rotated versions of the target. The second and third figures match the target figure in this example.

Participants were given the Audio‐Corsi task developed by Setti et al. (2021); see Figure 1 and the MRT (Figure 2). The Audio‐Corsi task involved the use of an Arduino‐based keyboard, which was supported by MATLAB software. The keyboard was made of a wooden box with six red buttons and a reference point (blue button) on the surface (Figure 1a). The keyboard was connected to the computer via USB. The red buttons on the surface of the keyboard replicated the six spatialized sounds. The sounds were delivered binaurally through headphones. All the sounds were the same stimuli (pink noise) for each location. The binaural spatialization of the sounds was supported by the 3D Tune‐In Toolkit Binaural Test App (Figure 1b; Cuevas‐Rodríguez et al. 2019). For more detailed information on the hardware, software, and the development of the Audio‐Corsi task, refer to Setti et al. (2021). The Audio‐Corsi task consisted of a maximum of 36 sequences for each of the two conditions (forward and backward). The first four sequences were made up of two sounds, the next four of three sounds, and so on. In each length of sounds, there were four sequences. The maximum length of sounds that could be reached was nine. The forward condition required the participant to recall the sound sequences in the same order of presentation, whereas the backward condition required them to recall them in reverse order of presentation.

#### 2.2.2 Mental Rotation

The MRT was composed of two sets of 12 trials. The stimuli for each trial consisted of a target figure on the left and four figures on the right (Figure 2). The participant had to identify the two figures that matched the target (Peters et al. 1995).

### 2.3 Procedure

Participants were first asked to read and sign a consent form and a personal information form. The personal information form asked for their sex, age, and undergraduate degree program. The Audio‐Corsi and MRT tasks were counterbalanced in order of presentation. For the Audio‐Corsi, the keyboard was placed in front of the participant (Figure 1c). The keyboard was previously covered with a black tablecloth, so participants were not able to get any visual information of the keyboard before the task started. Once the participants were seated comfortably, they put on the blindfold and the headphones. Before starting the task, the experimenter explored the keyboard together with the participant, guiding their dominant hand around the edges of the box, this was done to provide them with information about the dimensions of the box. After, the experimenter guided their index finger to each of the six buttons and to the reference point. The experimenter then explained to them the task (i.e., “Each of the buttons corresponds to a sound that will come from those six different locations, the reference point represents your head, your job is to recall sequences of sounds of increasing length, beginning with two sounds and up to potentially nine sounds, the sequence length will increase depending on your performance”). Participants had a 3‐min training phase to haptically explore the keyboard and familiarize themselves with the location of the sounds. After the 3‐min period, the experimenter followed to the “check of sounds” phase, where the participant was randomly given one of the sounds and asked to press the corresponding button after the sound. This phase ended when the participant had identified the six spatialized sounds. The training and check of sounds phases aimed to diminish perceptual and localization errors that could affect performance in the task (Setti et al. 2021). The experimenter then proceeded with the first condition of the task, the forward condition. The forward condition finished when the participant did not correctly recall any of the four sequences within a length of sounds. The backward condition was always given after the forward condition, and like the forward condition, it finished when the participant could not correctly recall any of the four sequences for a given length.

For the MRT, participants were verbally instructed to choose the two out of the four figures that matched the target figure. They were given a 3‐min time limit (recorded using a stopwatch) for each set of 12 trials with a 3‐min rest between the two sets.

At the end of the tasks, participants were asked to fill out a strategy questionnaire. The strategy questionnaire consisted of six questions (refer to Appendix A to see the full questionnaire). One example of these questions was as follows: “Indicate if you found yourself creating an image or picture when completing the auditory task. For example, you formed a picture that represented the sequence.” Participants responded on a scale from one to five (1 being *did not use these cues* and 5 being *definitely used these cues*) to the six questions. Lastly, participants filled out a debriefing form where they indicated if the data collected from them could or could not be used for the study.

### 2.4 Data Analysis

Two dependent variables were recorded for each condition (forward and backward) of the Audio‐Corsi task: (1) the memory span or the length of the longest sequence correctly recalled (maximum score of 9) and (2) the number of sequences correctly recalled (maximum score of 36). A total score was also calculated; this was the product between the number of correct sequences and the memory span (Setti et al. 2021). The Audio‐Corsi task was analyzed with condition (forward and backward) by sex (female and male) repeated measures ANOVA, with sex (female and male) as a between‐participant factor. The MRT was scored by taking the sum of only the trials in which the two answers were correct, dividing by the maximum score of 24, and then multiplying by 100 (Peters et al. 1995). To investigate sex differences on the MRT task, an independent sample *t* test was performed.

To investigate the relationship between the Audio‐Corsi and MRT, multiple linear regression analyses (method stepwise) were conducted. First all participants were included in the analysis, and then, the analysis was conducted for each sex separately. In both cases, the dependent variable was the MRT score, and the total score forward and total score backward were the independent variables.

Sex differences on the strategy questionnaire were investigated with independent samples *t* tests. To examine a possible relationship between Audio‐Corsi performance and the six self‐reported strategies (see Appendix A), stepwise multiple linear regressions were conducted separately for each sex. The dependent variables were the total forward or the total backward scores of the Audio‐Corsi, with the six strategies as independent variables. The alpha level for all comparisons was 0.05. IBM SPSS Statistics (Version 29) was used for all analyses.

## 3 Results

### 3.1 Sex Differences

#### 3.1.1 Audio‐Corsi Task

**FIGURE 3:**
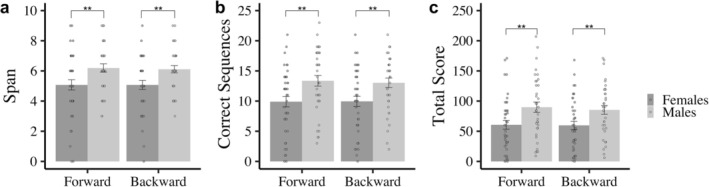
Performance of female and male participants in the Audio‐Corsi task. (a) Span, (b) sequences correctly recalled, and (c) total score. **p ≤ 0.01. Error bars represent standard errors.

Performance on the Audio‐Corsi task was analyzed with a condition (forward and backward) by sex (female and male) repeated measures ANOVA for the dependent variables. No significant main effect of condition was found. A main effect of sex was found in the number of correct sequences recalled in the forward and backward conditions, *F*_(1, 75)_ = 8.71, *p* < 0.01, $\eta_{p}^{2}$ = 0.10. Males correctly recalled more sequences than females did in both conditions (Figure 3a). Regarding the memory span (or length of the longest sequence correctly recalled), a significant main effect of sex was found, *F*_(1, 75)_ = 8.29, *p* < 0.01, $\eta_{p}^{2}$ = 0.10. Thus, males were able to recall more spatialized sounds in both, forward and backward conditions than females did (Figure 3b). As expected, the total score or the product of the two dependent variables also showed a significant main effect of sex: *F*_(1, 75)_ = 8.36, *p* < 0.01, $\eta_{p}^{2}$ = 0.10. Once again, total score was higher for males than females for both conditions (Figure 3c). No significant interaction of condition by sex was found in any of the dependent variables.

#### 3.1.2 Mental Rotation Task

Male participants (M = 44.9%, SD = 21.6) had better performance on the MRT than female participants (M = 30.0%, SD = 19.3), *t*_(73)_ = −3.2, *p* < 0.01, *d*_*s*_ = −0.73, [−1.20, −0.26]. Descriptives of the dependent variable of the MRT are shown in Table 1.

**TABLE 1:** Mean and standard error for the MRT values.

Dependent variable	Grand mean	Female participants	Male participants
MRT score (%)	36.9 ± 2.5	30.0 ± 3.0	44.8 ± 3.6

### 3.2 Audio‐Corsi and MRT Relationships

The multiple linear regression analysis with all participants was significant (*F*_[1, 74]_ = 11.38, *p* = 0.001, *R* ^2^ = 0.14; Table 2). The memory span forward was the only predictor of performance in the MRT. Furthermore, the multiple linear regression analysis, when split by sex, was significant for the female participants (*F*_[1, 39]_ = 11.73, *p* = 0.001, *R* ^2^ = 0.24; Table 3) but not for male participants. The memory span forward was the predictor of performance in the MRT for females. This relationship was found to be reciprocal, regardless of the MRT being the dependent variable or the predictor of the Audio‐Corsi performance.

**TABLE 2:** Results of the regression analysis. Note the only significant predictor of the MRT performance was the memory span forward.

Independent variable	Unstandardized *B*	Coefficients standard error	Standardized coefficients beta	*t*	Sig
(Constant)	15.15	6.87		2.21	< 0.05
Span forward	3.93	1.17	0.37	3.37	0.001

**TABLE 3:** Results of the regression analysis for females. The only significant predictor of the MRT performance for females was the memory span forward.

Independent variable	Unstandardized *B*	Coefficients standard error	Standardized coefficients beta	*t*	Sig
(Constant)	8.40	6.86		1.23	0.23
Span forward	4.30	1.26	0.49	3.43	0.001

### 3.3 Strategy Questionnaire

#### 3.3.1 Sex Differences

Significant sex differences were found in question two and in question five of the strategy questionnaire. For question two (“Indicate if you found yourself creating an image or picture when completing the auditory task. For example, you formed a picture that represented the sequence”), *t*_(68)_ = −2.24, *p* < 0.05, *d*_*s*_ = −0.54, [−1.01, −0.06], and for Question 5 (i.e., “Indicate if you found yourself creating a ‘whole’ of the sequence when completing the auditory task. For example, you created a map, a story, an image, of the sequence”), *t*_(68)_ = −3.15, *p* < 0.01, *d*_*s*_ = −0.75, [−1.24, −0.27]. In both cases, males rated using these strategies higher than females did. No significant sex differences were found in the other four questions.

#### 3.3.2 Audio‐Corsi and Strategy Questionnaire Relationships

The multiple linear regression analysis was significant for the male participants in both the total score of the forward condition (*F*_[1, 33]_ = − 11.72, *p* < 0.05, *R* ^2^ = 0.37; Table 4) and the total score for the backward condition (*F*_[1, 33]_ = 11.71, *p* < 0.05, *R* ^2^ = 0.36; Table 5). The allocentric strategy (Question 4) was the only predictor of their performance in the forward condition, whereas the detail/piecemeal strategy (Question 6) was the only predictor of their performance in the backward condition. There were no significant predictors of the Audio‐Corsi performance for female participants.

**TABLE 4:** Results of the regression analysis for males in the forward condition of the Audio‐Corsi. The only significant predictor was the allocentric strategy (Question 4).

Independent variable	Unstandardized *B*	Coefficients standard error	Standardized coefficients beta	*t*	Sig
(Constant)	127.84	19.04		6.72	0.00
Allocentric	−11.72	5.20	−0.37	−2.26	0.03

**TABLE 5:** Results of the regression analysis for males in the backward condition of the Audio‐Corsi. The only significant predictor was the detail/piecemeal strategy (Question 6).

Independent variable	Unstandardized *B*	Coefficients standard error	Standardized coefficients beta	*t*	Sig
(Constant)	43.29	21.14		2.05	0.05
Detail	11.71	5.42	0.36	2.16	0.04

## 4 Discussion

Sex differences in spatial abilities are well established in the literature, with males consistently outperforming females on most standardized measures. For example, visuospatial tasks such as the CBT and MRT demonstrate a male advantage (for meta‐analyses, see Voyer et al. 1995, 2017). However, despite extensive research on visuospatial abilities, there remains a significant gap in understanding sex differences in other sensory domains. The present study sought to address this gap by employing the Audio‐Corsi Task, an auditory version of the CBT (Setti et al. 2021) to investigate (1) sex differences in auditory‐spatial abilities, (2) potential relationships between visuospatial and auditory‐spatial performance in both females and males, and (3) whether sex‐specific cognitive strategies are utilized in solving auditory‐spatial tasks. We hypothesized a male advantage in auditory spatial abilities, a positive relationship between the visuospatial and the auditory‐spatial tasks in females and males, and a sex difference in cognitive strategy, respectively.

The results of this study provide compelling evidence of sex differences in auditory spatial cognition. Male outperformed female participants across all dependent variables of the Audio‐Corsi Task, including the number of correctly recalled sequences, memory span, and total score, thereby supporting our first hypothesis. These findings are consistent with previous research indicating a male advantage in visuospatial tasks, including the CBT (Voyer et al. 2017), and in tasks requiring auditory localization of spatialized stimuli (Lewald 2004). To our knowledge, this is the first study to demonstrate that the male advantage extends to spatial memory in the auditory domain.

Additionally, our results confirmed that the male advantage in spatial ability spans both auditory and visual domains within the same participant sample. Males in the current study scored better in the MRT than females. Confirming our second hypothesis, a significant relationship between MRT and Audio‐Corsi performance was observed with all participants together. However, a closer look at the data indicated that this relationship was only significant for female participants; the regression model for males was not significant. This finding is perplexing given that males showed a consistent advantage in both the auditory and visuospatial tasks. One might have expected that if performance on these tasks were related, the effect would be observed in both females and males. Other studies have demonstrated that when participants perform well in the MRT, they also perform well in other visuospatial tasks, some of which include the Rod‐and‐Frame, Water Level Test, and CBT (Quaiser‐Pohl et al. 2004; Garg et al. 2024). However, these studies report only the combined results of both female and male participants. Our findings suggest that greater attention should be given when examining the relationships between spatial tests as these relationships may be sex‐specific. Additionally, our results suggest that, in males, associations between spatial tasks may be confined to the same sensory modality whereas, in females, this association may extend across different sensory modalities. Evidence suggests that females and males differ in how they integrate visual and auditory stimuli. For instance, females exhibit stronger audiovisual integration than males in the McGurk effect—a perceptual phenomenon where conflicting auditory and visual information results in a fused or altered perception of speech (McGurk and MacDonald 1976; Irwin et al. 2006). This sex‐specific pattern of audiovisual integration may help explain the findings of the current study.

The findings of the current study may also be attributed to differences in how male and female brain networks process spatial information (Gur et al. 2000; Haier et al. 2005; Yuan et al. 2019; Ramos‐Loyo et al. 2022; Zhang et al. 2024). Zhang et al. used a graph‐theoretical, network‐based approach to probe the neural basis of sex differences in mental rotation. They found that, relative to males, females displayed stronger internetwork connectivity between the low‐level visual network and two high‐order systems, the fronto‐parietal and dorsal‐attention networks. The authors argue that this pattern indicates that females recruit low‐level visual perception more heavily to support demanding higher‐order operations. Males, in contrast, exhibited weaker visual network–high‐order coupling, which they interpret as reduced competitive interference, greater functional specialization, and thus superior accuracy on mental rotation. Given these findings and the results of the current investigation, we speculate that males may possess an integrated and specialized brain network for processing spatial information, that is, both sensory‐ and task‐specific. This could account for males' superior performance on most spatial tasks and the lack of correlation between tasks across different sensory modalities (i.e., the current result). In contrast, females may rely on a more distributed brain network for spatial processing, one that is less specialized and not tied to a specific sensory modality or task. This latter speculation warrants empirical testing, particularly through neuroimaging and cross‐modal task paradigms, to determine whether such integrated or distributed processing is indeed a characteristic feature of spatial cognition in males and females. If supported, this could explain the observed relationships between spatial tasks within sensory modality (Kaufman 2007; Garg et al. 2024) and, as in the current study, across visual and auditory modalities in females. Some existing evidence supports our speculation. For example, sex differences are found in the structural connectome of the human brain: Males exhibit higher modularity (i.e., within region connectivity) and intrahemispheric (i.e., within hemisphere) connectivity, whereas females show greater intermodularity (i.e., between region connectivity) and interhemispheric (between hemisphere) connectivity (Ingalhalikar et al. 2014; Tunç et al. 2016; Gur and Gur 2017). These structural differences have been linked to sex differences in spatial and other cognitive tasks (Ingalhalikar et al. 2014; Gur and Gur 2017). Furthermore, functional evidence has revealed stronger right‐hemisphere activation in males during spatial tasks, whereas females do not show a hemispheric preference (Gur et al. 2000; Vogel et al. 2003; Rilea et al. 2004). To date, no studies have paired behavioral and imaging data to investigate sex differences in brain networks while comparing spatial working memory across sensory modalities. This gap warrants further exploration.

An alternative nonmutually exclusive explanation involves differences in the engagement of specialized brain regions for spatial processing. Although females and males activate overlapping brain areas during spatial tasks (Hugdahl et al. 2006), males predominantly recruit specialized regions, such as the parietal cortex. In contrast, females engage prefrontal and other regions less directly associated with spatial processing (Weiss et al. 2003; Hugdahl et al. 2006; Zilles et al. 2016; Mayorova et al. 2023). This pattern suggests that females might rely on preferred, albeit less specialized, mechanisms to solve spatial tasks. For instance, females may prioritize working memory mechanisms over spatial ones, whereas males more effectively recruit spatial‐specific regions. Although this interpretation is conceptually supported by the previous imaging studies, it remains speculative and has yet to be directly tested, particularly in tasks across different sensory modalities. Future studies could continue to develop innovative approaches to test this hypothesis for other visual and nonvisual spatial tasks. Nonetheless, these differences in brain networks suggest that females and males might employ distinct strategies to perform similar cognitive functions; this is a concept explored in this study through self‐reported questionnaires.

*T*‐test analyses examining sex differences in strategies used for the Audio‐Corsi task revealed that male participants reported significantly greater reliance on imagery‐based and holistic strategies, such as forming a mental image or spatial map to represent auditory sequences. Although female participants also reported using these strategies, their preference was not significantly stronger than other strategies nor did they report a notable use of analytical or detail‐oriented approaches. Additionally, both sexes identified verbal strategies as the least used for this auditory spatial task. These findings are consistent with prior behavioral research demonstrating that males and females often employ different cognitive strategies when engaging in spatial tasks (Pletzer 2014; Saucier et al. 2002). For instance, in navigation tasks, males tend to rely more on allocentric cues (e.g., cardinal directions), whereas females often prefer egocentric cues (e.g., left/right). Similarly, in mental rotation tasks, males typically use a holistic approach (rotating the entire object), whereas females more often adopt a segmentary or piecemeal method (rotating parts individually). Notably, holistic strategies are generally linked to better task performance (Pletzer 2014). The current *t*‐test results extend these sex‐based strategic differences to the auditory spatial domain, supporting the notion that males prefer holistic or global strategies even when visual cues are absent. However, contrary to findings in visual spatial tasks, we found no evidence that females favored an egocentric or detail‐focused strategy more than males in the auditory version of the task. Nor did females report increased use of verbal strategies, which has been previously observed in visual spatial tasks (Ramos‐Loyo and Sanchez‐Loyo 2011; Merrill et al. 2016).

When exploring the relationship between strategy use and task performance by sex, multiple regression analyses revealed interesting results. For males, results aligned with existing literature: They tended to use an allocentric (global) strategy during the forward condition of the Audio‐Corsi task. However, unexpectedly, they adopted a piecemeal (segmentary) strategy in the backward condition. This shift may reflect fundamental differences in the cognitive demands of the two conditions. Although both require spatial processing, some studies have proposed that the backward condition is distinct in requiring sequential processing, that is, recalling each item in order rather than as a whole (Higo et al. 2014). This would make a piecemeal strategy more effective in the backward condition. These findings suggest that males may flexibly adopt the most efficient strategy depending on the task demands. Surprisingly, no relationship was found between strategy use and performance on the auditory task in females. This may suggest that females are not relying on a single dominant strategy but rather shift strategies throughout the task. These findings align with previous research suggesting that females do not always select the most efficient strategy when solving spatial tasks (Piber et al. 2018). To better understand these patterns, future studies should use objective measures (rather than self‐reported questionnaires) to assess whether differences in cognitive strategy mediate performance on auditory and visual spatial tasks in females and males.

It is important to note that this is the first study to investigate sex differences in spatial working memory using auditory stimuli. As such, further research is necessary to replicate and extend these findings. Two key limitations should be acknowledged. First, the study did not include a purely visuospatial working memory task (e.g., the Corsi Block Taping test), which would have allowed for direct comparisons between auditory‐spatial and visuospatial processing. To address this, we are currently conducting a follow‐up study that includes a purely visuospatial working memory task, enabling more comprehensive cross‐modal comparisons. Second, although the current sample is adequately powered to detect overall sex differences, it is smaller than those used in recent large‐scale studies (e.g., Tsigeman et al. 2023) and meta‐analyses (e.g., Xu et al. 2017), limiting our ability to detect more subtle interaction effects such as those involving age. Additionally, the current implementation of the Audio‐Corsi task restricts scalability; however, a computerized version could facilitate testing with larger and more diverse samples, a promising direction for future research. Given the limited research in this area, we speculated on differences in the underlying mechanisms that could have given rise to the results of the present study. However, future research should investigate the neural correlates of these observed sex differences by using neuroimaging techniques paired with behavioral spatial tasks (such as the Audio‐Corsi).

In conclusion, the present study examined sex differences in auditory spatial cognition. First, the findings revealed that males outperformed females in both auditory and visuospatial tasks. Second, a relationship between auditory and visuospatial tasks was observed in females but not in males. Third, males reported using holistic strategies to solve the auditory task, whereas females showed no preference. The male advantage in both auditory spatial working memory and mental rotation tasks, coupled with the sex‐specific relationship between auditory working memory and mental rotation, may reflect differences in the organization or functioning of spatial networks in the brain. This study contributes to a deeper understanding of sex differences in spatial cognition and begins to address a significant gap in the literature. From a practical perspective, these findings have important implications for educational and professional settings. The development of multisensory tasks that engage both auditory and visual spatial systems may enhance spatial learning, particularly for females, by leveraging their tendency to integrate information across modalities. As our understanding of sex differences deepens, such targeted, multimodal training approaches could help reduce disparities in spatial cognition and improve educational outcomes in STEM and other spatially demanding domains.

## Author Contributions

**Daniela E. Aguilar Ramirez:** data curation, formal analysis, methodology, project administration, visualization, writing – original draft, writing – review and editing. **Jennifer Kane:** data curation. **Walter Setti:** methodology, software, writing – review and editing. **Lara Coelho:** methodology, writing – review and editing. **Monica Gori:** writing – review and editing. **Claudia L. R. Gonzalez:** funding acquisition, methodology, supervision, writing – review and editing.

## Ethics Statement

All procedures performed in studies involving human participants were in accordance with the ethical standards of the institutional and/or national research committee and with the 1964 Helsinki declaration and its later amendments or comparable ethical standards.

## Consent

Informed consent was obtained from all individual participants included in the study.

## Conflicts of Interest

The authors declare no conflicts of interest.

## Peer Review

The peer review history for this article is available at https://www.webofscience.com/api/gateway/wos/peer‐review/10.1111/ejn.70226.

## Data Availability

The materials and data that support the findings of this study are available through Mendeley Data (https://data.mendeley.com/preview/84dvn372n3?a=ad27a028‐9806‐4902‐a848‐e173c042132c).
